# Lessons Learned from Surgical Management of the Largest Burned Patient Covered with Skin Isograft from His Monozygotic Twin Brother

**DOI:** 10.3390/ebj7010013

**Published:** 2026-02-21

**Authors:** Maurice Mimoun, Marc Chaouat, Nathaniel Malca, Oren Marco, David Boccara, Kevin Serror

**Affiliations:** 1 Faculty of Medecine, Université Paris Cité, 75006 Paris, France; 2 Department of Plastic Surgery and Burn Center, Saint Louis Hospital, APHP, 75010 Paris, France

**Keywords:** isograft, twin, monozygotic, monozygotic, extended burns

## Abstract

**Background:** Early excision and autologous split-thickness skin grafting are the cornerstone of surgical management in severe burn injuries. In patients with extremely extensive deep burns, the lack of available donor sites represents a major life-threatening limitation. In the exceptional situation of monozygotic twins, skin isografting offers a unique solution by providing immunologically compatible skin without the risk of rejection. **Case report:** We report the case of a 33-year-old man who sustained flame burns involving 95% of his total body surface area, resulting in an extremely poor initial prognosis (ABSI 14, UBS 245). After early resuscitation and staged surgical excisions, the absence of sufficient autologous donor sites precluded definitive coverage using conventional techniques. On day 3, the existence of a monozygotic twin brother was identified. HLA genotyping confirmed complete identity, and skin donation was authorized by an independent ethics committee. **Methods:** Definitive wound coverage was achieved using staged split-thickness skin isografts harvested from the donor twin. Ultra-thin grafts (<0.2 mm) were obtained in three procedures (days 7, 11, and 45), primarily from the scalp, thighs, and back, and applied following sequential excisions. **Results:** All grafts survived without immunological rejection. Donor-site morbidity was minimal, with rapid healing and only mild residual hypopigmentation. The patient was discharged to rehabilitation on day 145. At 5-year follow-up, wounds were fully healed, functional outcome was satisfactory, and quality of life was good, with return to work and full independence. **Conclusions:** Skin isografting from a monozygotic twin is a rare but effective salvage strategy for patients with massive deep burns when autologous donor sites are insufficient, provided that ethical, legal, and donor safety considerations are rigorously addressed.

## 1. Introduction

Surgical management of patients with severe burns relies on early excision of the necrotic tissues followed by definitive coverage with autologous split-thickness skin grafts harvested from uninjured donor sites. Early excision and autografting have been shown to significantly reduce mortality, infectious complications, and length of hospital stay in extensively burned patients [1,2]. However, in cases of large-surface deep burns, the availability of suitable donor sites rapidly becomes the main limiting factor for definitive wound coverage.

When donor sites are insufficient, the patient’s prognosis is compromised and achieving rapid and durable skin coverage becomes a major therapeutic challenge. To address this issue, various surgical methods have been developed to maximize the surface covered with limited autologous skin, such as: postage stamp grafting, mesh grafting [3], intermingled auto- and allograft transplantation [4], associated auto- and homografts, microskin grafting, Meek technique [5] or even cultured keratinocyte transplantation. These techniques are often associated with prolonged healing process, increased risk of systemic complications, and frequently result in suboptimal functional and aesthetic outcomes, particularly in patients with very extensive full-thickness burns.

In the exceptional situation of monozygotic twins, skin isografting represents a unique alternative, combining the immunological advantages of autografting with the availability of an additional donor surface. Owing to their identical genetic background, isografts from a monozygotic twin are not subject to immune rejection and may provide immediate, definitive coverage without the need for immunosuppression. Nevertheless, reports of twin skin isografting in burn care remain exceedingly rare, and its indications, feasibility, and practical management are not well defined.

The purpose of this article is to report the case of the most extended deep burned patient successfully treated with split-thickness skin isografts harvested from his monozygotic twin brother ever reported in the literature, to review the existing literature and to discuss the indications, surgical management, ethical considerations, and outcomes of skin isografting in severely burned patients.

## 2. Methods

The patient was managed in our burn center according to the standard institutional protocols for the treatment of severe burns. Clinical data, operative details, and photographic documentation were prospectively collected and retrieved from the patient’s electronic medical record. The manuscript was prepared and submitted with the patient’s written informed consent, including consent for the use of clinical photographs.

In addition to the case report, a literature review was performed using the PubMed database. The search strategy combined the following keywords: “isograft”, “twin”, “burns”, and “monozygotic”. Relevant articles describing the use of skin isografts from monozygotic twins in burn patients were identified and reviewed. Epidemiological data, surgical indications, grafting techniques, and reported outcomes from the retrieved studies were analyzed and are presented in the Discussion section.

## 3. Results—Case Report

**Figure 1:**
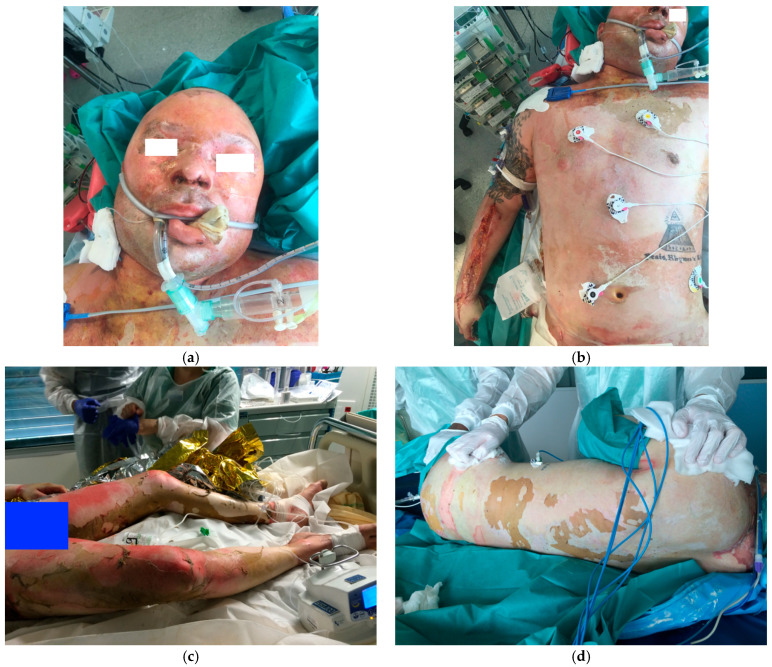
Patient at his admission (day of the accident): Burns of the face (**a**), Trunk (**b**), Lower limbs (**c**) and back (**d**).

A 33-year-old man was admitted to our burn center with flame burns involving 95% of the total body surface area (TBSA), sustained during an industrial accident at a hydrocarbon plant. The only unburned areas were the feet, right shoulder, external genitalia, and a small inguinal fold. Regarding the burns, 55% were third-degree, 30% were intermediate and deep second-degree, and 10% were more superficial burns (Figure 1a–d). Burn severity scores indicated an extremely poor prognosis, with a Unified Burn Severity (UBS) score of 245 and an Abbreviated Burn Severity Index (ABSI) of 14 [6].

On day 0, within a few hours of injury, escharotomy and releasing incisions were performed on all four limbs and the chest. Initial wound management consisted of topical Flammacerium^®^ (silver sulfadiazine 1% combined with cerium nitrate 2.2%).

On day 1, tangential excision of the left thigh and leg was performed. Wound coverage was achieved using micro-autografts harvested from the inguinal region and applied to the deep surface of cadaveric allograft. Due to the scarcity of donor sites, 1 mm^2^ micro-autografts were applied to the deep surface of the allografts to promote localized epidermalization. This method was chosen as a more skin-sparing alternative to the Meek technique, allowing conservation of autograft tissue during the acute phase while establishing multiple small foci of epidermal growth beneath the allograft. The patient underwent tracheostomy the same day.

On day 2, excision of the contralateral thigh and leg was carried out using the same technique.

On day 3, it was discovered that the patient had a twin brother who declared himself to be monozygotic and expressed a strong willingness to donate skin. After detailed information regarding the risks, potential sequelae, and donor-site morbidity associated with split-thickness skin graft harvesting under general anesthesia, the twin brother reaffirmed his consent. Human leukocyte antigen (HLA) genotyping confirmed complete identity between the two brothers, supporting monozygosity and identity at the major histocompatibility complex (MHC) loci. An independent ethics committee convened an emergency meeting and, after direct consultation with the potential donor, granted authorization for skin isografting.

The first Isografting procedure was performed on day 7. Two surgical teams worked simultaneously in adjacent operating rooms: one team performed burn excision on the recipient, while the other harvested split-thickness skin grafts from the donor. Skin was harvested from the scalp and right thigh of the donor following infiltration with an adrenaline-containing solution (1 mg adrenaline per liter of isotonic saline combined with 75 mg ropivacaine). Particular attention was paid to harvesting very thin grafts to minimize donor-site morbidity. An Acculan^®^ 3Ti dermatome (B. Braun SE, Melsungen, Germany) was used to harvest grafts with a thickness of 0.2 mm. The grafts were immediately applied to the recipient’s hands as sheet grafts and meshed for coverage of the left upper limb and ipsilateral hemithorax.

A second isografting procedure was carried out on day 11 following the same protocol. Grafts were harvested from the donor’s back and contralateral thigh and applied to the recipient’s right upper limb, contralateral hemithorax, and neck. At the end of this procedure, all burned areas had been excised and covered.

**Figure 2:**
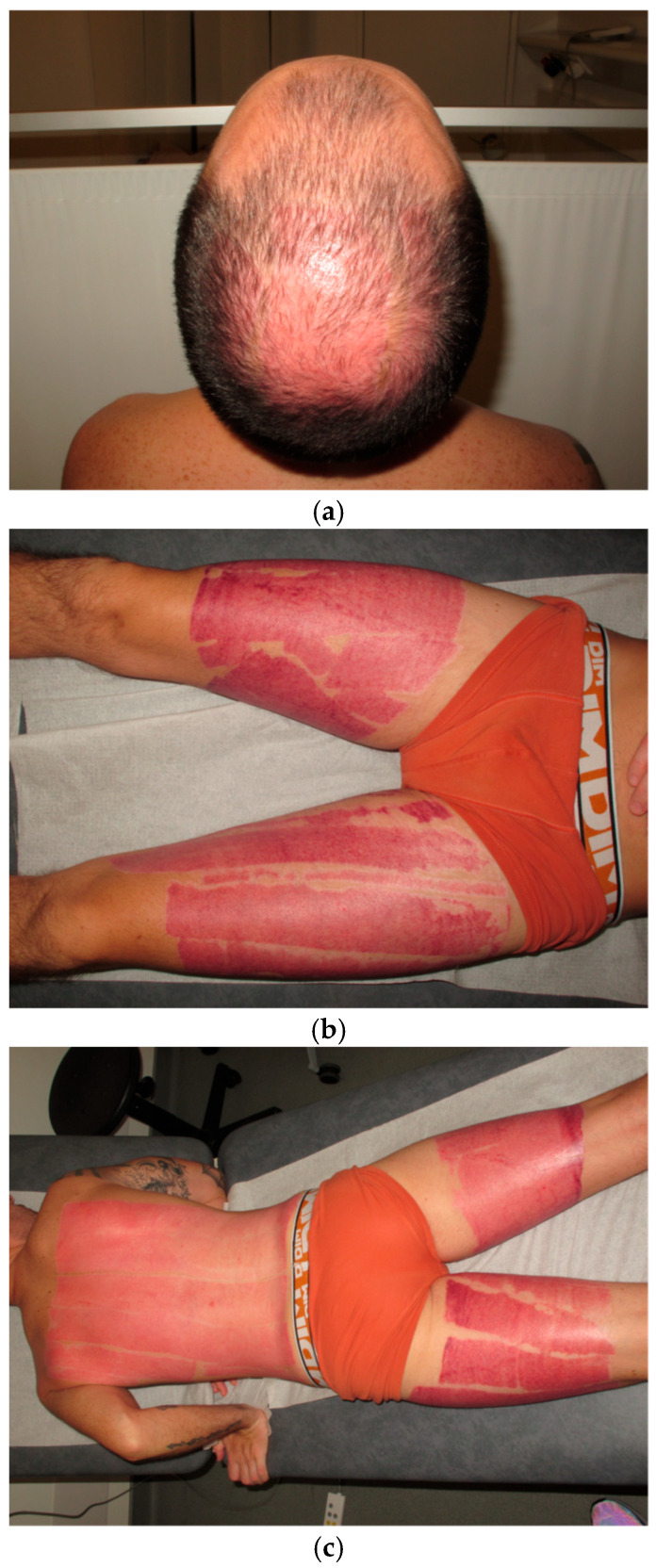
Aspect of the donor sites 30 days after surgery Scalp donor site was completely healed (**a**), back (**b**), and thigh (**c**) remains erythematous.

Concerning the harvesting of the isografts, a donor-conservative approach was employed, including the use of thin grafts, appropriate dressings, and compression garments to minimize the risk of hypertrophy in the healthy donor. By day 30, the donor had achieved complete epithelialization of all donor sites and no longer required dressings (Figure 2a–c).

On day 45, a third isografting procedure was performed, with grafts harvested from the donor’s scalp and used to provide complementary coverage of the recipient’s buttocks and back. In total, the patient underwent excision over 80% of TBSA and was grafted with meshed skin isografts (ratio x3 on the upper limbs and x4 on the lower limbs and trunk).

The patient was progressively weaned from mechanical ventilation and was liberated from ventilatory support on day 82. Decannulation was performed on day 97. On day 145, he was discharged to a rehabilitation center with near-complete wound healing. He remained in inpatient rehabilitation for 5.5 months, followed by 9.5 months of outpatient rehabilitation and one additional year of home-based therapy. He returned to work 2.5 years after the accident.

The donor brother was hospitalized for four days for each of the first two isografting procedures and for two days following the third. Donor sites healed spontaneously without pathological scarring, within 4–5 days for the scalp and approximately 10 days for other donor areas. He resumed professional activity 2.5 months after the last harvesting procedure.

### Outcomes

**Figure 3:**
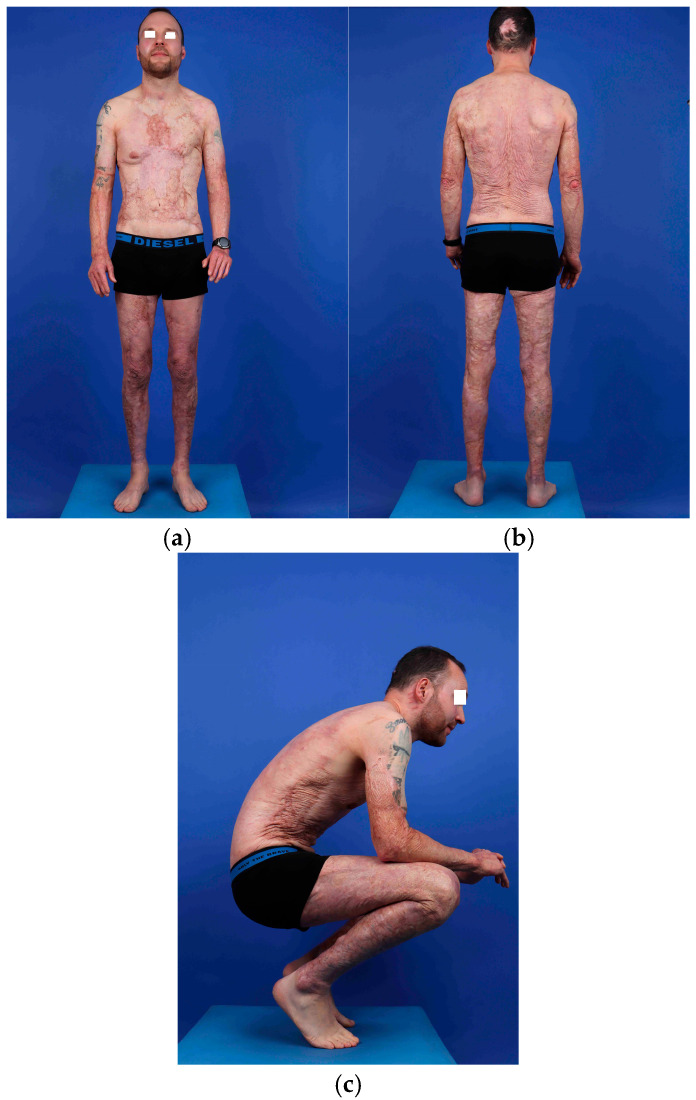
Appearance of skin isografts at 5 years, front (**a**) and back (**b**) views, in standing and squatting positions (**c**).

At 9 months post-injury, all grafted areas had healed, although some fragile zones persisted, particularly on the back, with recurrent superficial ulcerations. These areas stabilized within one year. At 5-year follow-up, the patient exhibited minimal sequelae on the face and scalp, and stable scars over the remainder of the body. Joint mobility was moderately reduced but compatible with full independence in activities of daily living. The patient reported the ability to jog 5 km daily (Figure 3a–c).

**Figure 4:**
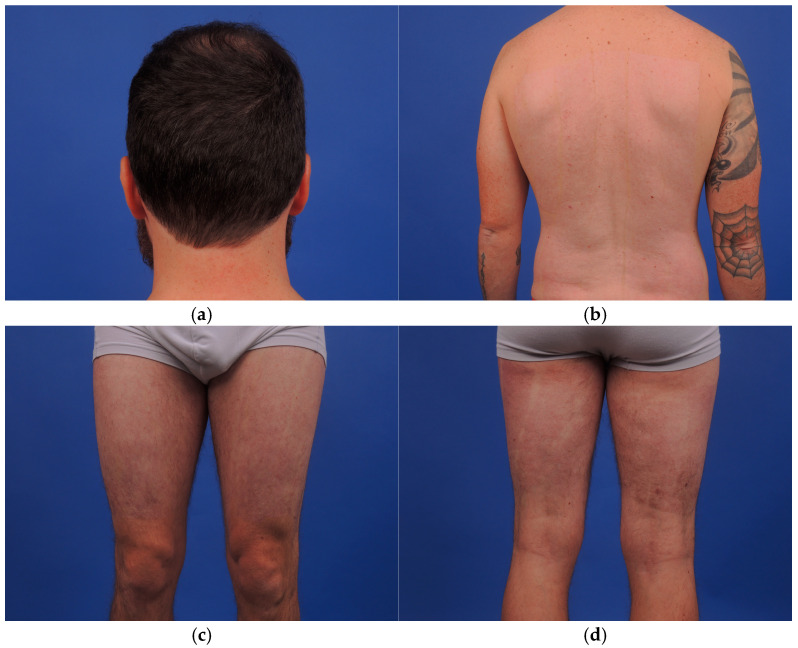
The donor sites on the brother healed without pathologic scars at 15 months. The sequelae are minimal or nil on the skull where the hair has grown back (**a**), and a mild hypopigmentation remained on the back (**b**) and lower limbs (**c**,**d**).

At one year, the donor brother presented only mild hypopigmentation at donor sites, without functional limitation or pain (Figure 4a–d).

## 4. Discussion

This report described, to our knowledge, the most extensively burned patient ever successfully treated using split-thickness skin isografts harvested from a monozygotic twin. In this exceptional clinical situation, twin isografting played a decisive role in achieving definitive wound coverage and patient survival.

The surgical strategy relied on staged excisions [7,8], limiting each procedure to a maximum of 20% TBSA and an operating time of no more than two hours. This approach aimed to reduce blood loss, hemodynamic instability, and physiological stress, which are known to significantly impact outcomes in severely burned patients. Survival in this case was made possible only through the close integration of customized surgical management and advanced burn critical care. While essential, the specific intensive care strategies employed fall beyond the scope of the present report.

Historical reports have explored skin transplantation between related individuals to overcome donor-site limitations. Peer et Al. [9] described full-thickness skin grafts performed between 31 ABO-compatible parents and infants, noting longer survival of maternal homografts compared with paternal grafts. In one remarkable case, maternal skin transplanted to an infant survived for eight months [10]. These observations supported the antigen–antibody theory of homograft rejection and suggested that prolonged graft survival could result from partial immune tolerance acquired through maternofetal blood cell exchange during pregnancy. However, such tolerance was never sufficient to allow definitive graft take.

Similarly, Bishop [11] reported cross-grafting between two dizygotic twin sisters with closely related but distinct blood group antigens (O, N, S+, CDe/cDE (R1R2), K+, Fya- and O, M, S+, CDe/cDE (R1R2), K-, Fya+). Despite an initial tolerance period of four weeks, graft rejection ultimately occurred, necessitating autologous split-thickness skin grafting. These cases underline the fundamental immunological barrier to definitive homografting outside monozygotic twin pairs.

Since Bauer’s first description of successful skin grafting between identical twins in burn care in 1927 [12], we identified 17 published reports of twin isografting between 1937 and 1996 [12,13,14,15,16,17,18,19,20,21,22,23,24,25,26,27,28]. Collectively, these publications indicate that skin donation from a monozygotic twin can favorably influence survival and outcomes in extensively burned patients. Most reported cases involved children or young adults. Only two reports described patients aged 60 and 65 years with approximately 50% TBSA burns [25,27]. In older donors, the risks associated with general anesthesia and skin harvesting must be carefully weighed against the increased mortality risk of the burned recipient. In these reported cases, isografting was limited to a single procedure covering no more than 20% TBSA to minimize donor risk.

Across the literature, the mean reported surface area of deep burns treated with isografts was approximately 45% TBSA (range: 6–68%). In more than half of the cases, burn size did not exceed 50% TBSA. While some authors define extensive burns as those exceeding 50% TBSA [29,30], others consider 60% TBSA as the upper limit for the effective use of widely meshed autografts [31]. Based on our experience, we suggest that isografting should primarily be considered for patients with deep burns exceeding 70% TBSA, in whom conventional autologous split-thickness skin grafting alone is insufficient to achieve timely and durable coverage. No complications have been reported to date in the literature for the donor.

Importantly, no significant donor complications have been reported in the literature to date. In our case, approval was obtained from an independent ethics committee, which concluded—after direct consultation with the donor—that the expected benefit justified the procedure [32]. We advocate that burn unit directors proactively address the possibility of urgent skin harvesting from monozygotic twins within their institutional and national legal frameworks. In France, a decree authorizing skin graft harvesting from living monozygotic twin donors was issued on 17 February 2022, partly as a consequence of this clinical experience. We deliberately awaited publication of this decree before submitting the present case to ensure full ethical compliance [33].

The incidence of monozygotic twinning is estimated at 3.5–4.5 per 1000 births in Europe [27]. Therefore, the initial evaluation of any patient with extensive deep burns should systematically include inquiry into the possible existence of a twin sibling. This information may not be immediately disclosed by families during the acute shock phase, as illustrated by our case, in which the presence of a twin brother was only identified on day 3.

Our experience demonstrates that repeated skin harvesting from the donor is feasible and safe. In this case, grafts were harvested on days 7, 11, and 45. Donor-site morbidity was minimal and limited to mild hypopigmentation. This favorable outcome was achieved by harvesting ultra-thin grafts (0.2 mm). The scalp should be considered the preferred donor site, as it heals rapidly (4–5 days versus 10–12 days for other sites), can be reharvested multiple times after complete epithelialization, and carries a very low risk of pathological scarring or complications [34,35,36,37,38,39,40]. The back and thighs, which are easily concealed by clothing, represent appropriate secondary donor sites when large surface areas are required urgently. Other areas—such as the upper limbs, shoulders, legs, face, neck, thorax, and abdomen—should ideally be spared in healthy donors to avoid functional impairment or visible scarring.

Cultured autologous keratinocytes represent an alternative strategy in patients with extensive deep burns [41]. Cuono proposed their use primarily to accelerate donor-site healing and to cover deep partial-thickness burns, often in combination with widely meshed split-thickness skin grafts [42,43,44]. However, due to their single-cell-layer structure and poor take on full-thickness wound beds, keratinocyte cultures are unsuitable for definitive coverage of third-degree burns. Additionally, the required culture period of approximately three weeks necessitates either delayed excision or prolonged temporary coverage with allografts or xenografts, both of which are associated with increased risks of infection, scar contracture, and inferior functional and aesthetic outcomes.

At five-year follow-up, the quality of life of our patient was good. He returned to work, married, and became a father four years after the injury. From a psychological standpoint, the donor twin expressed profound satisfaction with his contribution, emphasizing the importance of considering both physical and psychosocial outcomes when evaluating the impact of twin isografting.

## 5. Conclusions

This case represents, to our knowledge, the most extensive burn successfully treated with skin isografts harvested from a monozygotic twin reported in the literature. Early identification of a monozygotic twin is crucial, as the availability of a genetically identical donor can profoundly modify the prognosis of patients with massive deep burns.

The successful survival of a patient with 95% TBSA burns highlights that, beyond advances in critical care, the principal limiting factor in the management of extreme burns remains the availability of sufficient skin capable of providing definitive coverage without immune rejection. In this context, twin isografting constitutes a rare but highly effective salvage strategy when performed within a rigorous ethical and legal framework and with careful attention to donor safety. Repeated harvesting of ultra-thin split-thickness grafts, particularly from the scalp, can be achieved with minimal donor-site morbidity. Moreover, this case highlights the need for dedicated registries and evidence-based guidelines to better address the ethical and clinical complexities associated with twin isografting.

Given the rarity of monozygotic twins, this experience underscores the need to continue developing alternative solutions to overcome the shortage of immunologically compatible skin, including improved skin substitutes, accelerated cell culture technologies, and approaches aimed at inducing or reproducing graft tolerance.

## Data Availability

The data presented in this study are available upon request from the corresponding author due to privacy restriction.
